# Gas Sensors Based on Single-Wall Carbon Nanotubes

**DOI:** 10.3390/molecules27175381

**Published:** 2022-08-24

**Authors:** Shu-Yu Guo, Peng-Xiang Hou, Feng Zhang, Chang Liu, Hui-Ming Cheng

**Affiliations:** 1 School of Materials Science and Engineering, Northeastern University, Shenyang 110819, China; 2 Shenyang National Laboratory for Materials Science, Institute of Metal Research, Chinese Academy of Sciences, Shenyang 110016, China

**Keywords:** single-wall carbon nanotubes, gas sensor

## Abstract

Single-wall carbon nanotubes (SWCNTs) have a high aspect ratio, large surface area, good stability and unique metallic or semiconducting electrical conductivity, they are therefore considered a promising candidate for the fabrication of flexible gas sensors that are expected to be used in the Internet of Things and various portable and wearable electronics. In this review, we first introduce the sensing mechanism of SWCNTs and the typical structure and key parameters of SWCNT-based gas sensors. We then summarize research progress on the design, fabrication, and performance of SWCNT-based gas sensors. Finally, the principles and possible approaches to further improving the performance of SWCNT-based gas sensors are discussed.

## 1. Introduction

The detection of harmful gases and vapors is of great importance in relation to national defense [1], monitoring environmental pollution [2,3,4] and industrial emissions [5,6], and medical diagnosis [7,8]. Especially, with the coming of the Internet of Things (IoT) era, the development of high-performance portable and wearable gas sensors able to work at room temperature has attracted great research interest. Single-wall carbon nanotubes (SWCNTs) composed of a single layer of sp^2^-hybridized covalently-bonded carbon atoms have a unique one-dimensional tubular structure, high specific surface area, and excellent mechanical, electrical, thermal, and chemical properties. They are therefore considered an ideal candidate for the fabrication of high-performance gas sensors. Traditional gas sensors are usually assembled using metal oxide semiconductors, which work well in a high temperature range of 150~400 °C but this may decrease their sensing stability and lifetime [9] and bring risks of combustion and explosion and high power consumption, which are undesirable for the next-generation portable and wearable gas sensors [10]. In contrast, CNTs have a high sensitivity for target analytes at room temperature [11], due to their enhanced adsorption rates of gases and vapors originating from their high surface area [12]. Since Dai and co-workers [13] first reported a chemical sensor fabricated using an individual CNT in 2000, notable progress has been made in the development of CNT-based gas sensors.

In this article, we first briefly introduce the geometric structure of SWCNTs and their unique physiochemical properties that are related to their use as sensors. We then summarize the working mechanism and principles, device structures and the figure of merit of SWCNT-based gas sensors. The key issues to be addressed in optimizing the performance of SWCNT-based gas sensors are discussed, and possible solutions and developing trends for next-generation sensors are suggested. The article presents an overview of progress in SWCNT-based gas sensor development and sheds light on the development of high-performance flexible gas sensors for use in the IoT and portable and wearable devices.

## 2. Working Mechanism of SWCNT-Based Gas Sensors

### 2.1. SWCNT Structure

**Figure 1:**
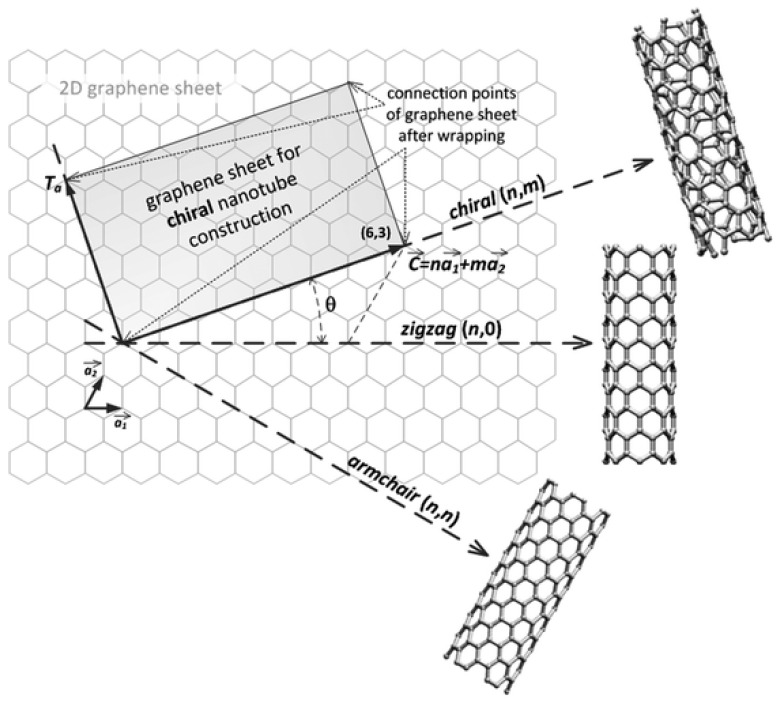
Schematic of SWCNTs rolled from graphene sheets using different chiral angles, Reproduced with permission from Royal Society of Chemistry [9].

The construction of an SWCNT can be conceptualized by rolling a perfect graphene sheet into a cylinder along the chiral vector $\vec{C}=n\vec{a_{1}}+m\vec{a_{2}}$ as shown in Figure 1 [9]. Three types of SWCNT can be formed according to roll-up vectors (*n*,*m*). The (*n*,0) structure is called “zigzag” and the structure where *n* = *m* (*n*,*n*) is called “armchair”. The third, where *n* > *m* > 0, is called “chiral”. The chirality determines the electrical, mechanical, optical, and other properties of SWCNTs. For example, an SWCNT can be either semiconducting (s-) or metallic (m-) depending on its chirality. Metallic and semi-metallic SWCNTs have roll-up vectors such that n - m=3q (where *q* is an integer) and semiconducting CNTs have n - m=3q ± 1. The distinction between semiconducting and metallic SWCNTs is important in the operation of nanotube-based field effect transistor (NTFET) devices [10]. The strong covalent carbon-carbon bonds make SWCNT a superb structural material with an ultrahigh stiffness (up to 1 TPa) and tensile strength (experimentally approaching 80 GPa [11]) in the direction of the tube axis. Meanwhile, the sp^2^ hybridization gives it fascinating electrical properties that depend on the diameter and helicity [12]. This combination leads to extraordinary mobility [13] and excellent quantum ballistic transport [14]. In addition, the large surface-to-volume ratio of SWCNTs, and their porous structure formed by interconnected tubes (or tube bundles) means that the carbon atoms are exposed to the environment and can be functionalized with abundant [15] and effective binding sites for gas molecules [16]. One of the most attractive features of SWCNT-based gas sensors is their ability to form flexible sensors for various gases [17,18,19], as well as working at room temperature with a low power consumption [20].

### 2.2. Sensing Principle

**Figure 2:**
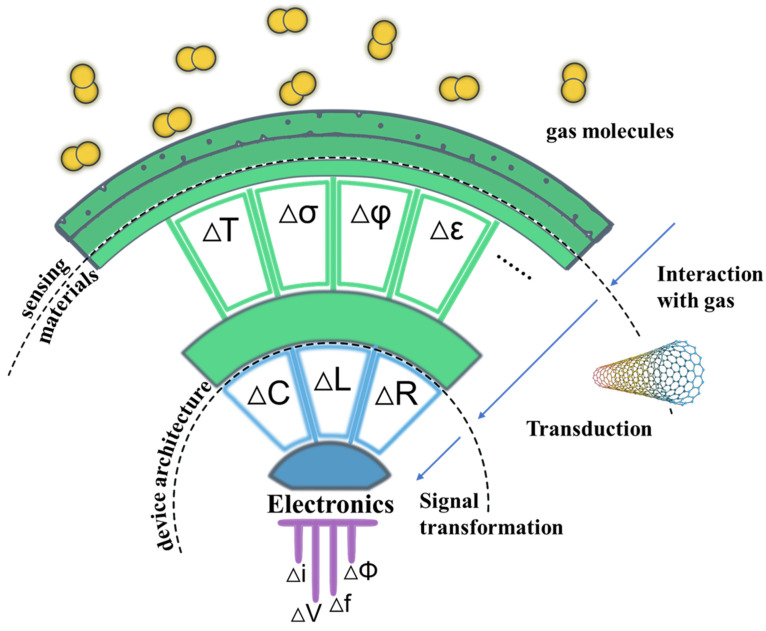
Logical structure of a gas sensor. Adapted with permission from American Chemical Society https://doi.org/10.1021/acs.chemrev.6b00361 (accessed on 4 August 2022) [28]. Analytes interact with the sensing material (CNTs or functional active sites on CNTs) changing some of its physical properties (e.g., temperature, ΔT; conductivity, Δσ; work function, ΔΦ; and permittivity, Δε). Transduction converts one of these physical quantities into a change in an electrical parameter (capacitance, inductance, and resistance are mentioned). Finally, the circuit connected to the sensor gives rise to a signal, usually a current or voltage change that can be measured.

According to the International Union of Pure and Applied Chemistry, a chemical sensor is defined as a device that transforms chemical information, ranging from the concentration of a specific sample component to total composition analysis, into an analytically useful signal [21]. Such devices are logically made up of two main components: the sensing material (or receptor) and the transducer (Figure 2). Pure SWCNTs alone act as both the sensing material and the transducer, directly recognizing gases or vapors such as NO_2_, NH_3_, benzene and benzene derivatives with high affinity and transducing them into measurable signals. In addition, SWCNTs are a superb medium for functionalization that can detect insensitive gases towards pristine CNTs. SWCNT-based gas sensors can be classified according to the type of signal they produce, either electrical, optical [22,23,24], capacitive [25] and acoustic [26]. Among these, sensors that produce electrical signals are preferred due to their simplicity, portability, compatibility with standard electronics, and ability to be continuously monitored [27]. This review mainly focuses on gas sensors that produce electrical signals.

### 2.3. Important Figure of Merit of Gas Sensors

**Figure 3:**
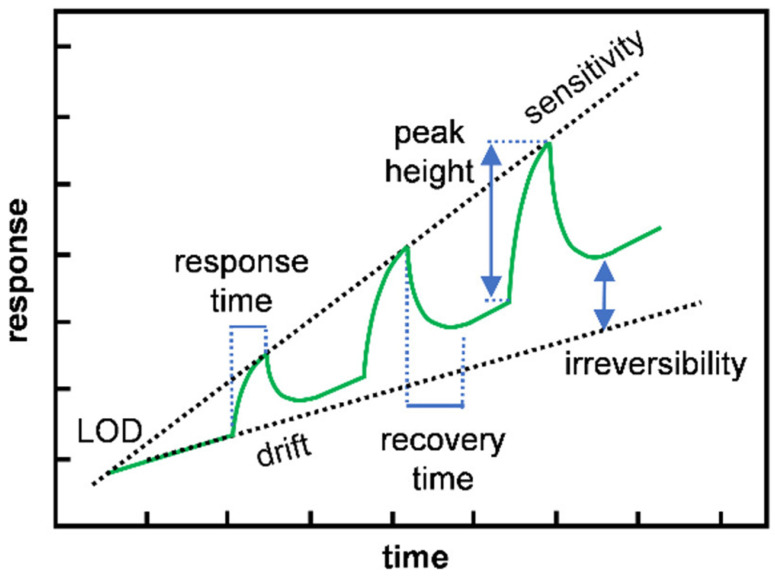
Graphical representation of several important performance parameters in a sensor exposed to increasing concentrations of analyte gas. Reprinted with permission from [30], Copyright 2019, American Chemical Society.

An ideal gas sensor needs to have the following features: (i) high sensitivity to low gas concentrations, (ii) rapid response, (iii) reversible operating ability, (iv) good selectivity to different gases of interest, (v) low-manufacturing cost, (vi) stable operation over many cycles of use, and (vii) low power consumption during operation [29]. Figure 3 shows a representative chemiresistive sensor upon three successive exposures to increasing analyte concentrations, that graphically represent the key features of the sensing performance, including sensitivity, limit of detection (LOD), response/recovery time, drift, and reversibility [30]. We now give a brief introduction to these performance parameters.

#### 2.3.1. Sensitivity

Sensitivity is defined as the ability to discriminate small differences in the concentration of the analyte gas, and it can be calculated by the relative changes in the signal measured by the sensors, including resistance, current, conductance, capacitance, and power gain, depending on the type of sensor. Taking a resistive sensor as an example, the change in resistance (ΔR/R_0_) is calculated by observing the resistance values before (R_0_) and after (R) exposure to the gas:

(1) $$\mathrm{Sensitivity}=\frac{R_{1}{-R}_{0}}{R_{0}}\times 100\%$$

A site-binding hypothesis assumes that atoms on the surface of the sensing material act as binding sites for analyte adsorption, and thus the conductance change of the device is related to the surface occupancy of the analyte molecules on the sensing materials [31]. The sensitivity is therefore improved by introducing binding sites for the analyte.

#### 2.3.2. Response Time and Recovery Time

The response and recovery times are important factors when evaluating the performance of a gas sensor. The response time is defined as the time for the sensor to reach 90% of its steady state or maximum value on exposure to a given concentration of the analyte, while the recovery time is the time taken to recover 90% of its peak value [32]. The response time is strongly dependent on the device structure, recognition components, and analytical techniques used to generate the signal [30]. A fast response time is desired for the real-time and continuous detection of gases and their monitoring [33,34]. The recovery time is considered as the converse of the response time. Generally, a fast response time is accompanied by a slow recovery time due to chemical adsorption. Because of this, treatments such as UV irradiation [35] and heating [36] are often used to improve the gas desorption behavior.

#### 2.3.3. Limit of Detection

LOD is the lowest concentration of target gas which can be reliably distinguished with a specified precision and reproducibility (typically with a 99% confidence level) [27]. The LOD of a sensor can be influenced by receptor–analyte interactions, surface area, functionalization, and signal amplification [37], and is closely tied to high sensitivity. The higher the affinity between target gas and SWCNTs (or functionalized SWCNTs), the lower the LOD, the faster the gas sensing response, and the harder the recovery of the sensors. To achieve rapid gas desorption, additional energy inputs such as heating or UV light irradiation are usually required to reactivate the sensors. The LOD of an SWCNT-based NO_2_ sensor has already reached the sub-ppb level [38], and an SWCNT-based sensor can even detect a single molecule [39,40,41] for some chemicals in the vapor phase.

#### 2.3.4. Drift

Drift is the slow, non-random change of signal with time while the concentration of the measured analyte remains constant. Although drift can be addressed either by in-device recalibration or algorithms during data processing and/or workup, many applications cannot sustain intensive computational solutions to sensor drift [37]. Drift is undesirable for practical sensors and remains a challenge to be solved for CNT-based sensors.

#### 2.3.5. Selectivity

Selectivity is the ability of a sensor to identify the target gas present in a sample containing several other interfering chemicals [21]. Although the selectivity of pristine SWCNTs is usually poor due to their robust and stable C-C covalent bonding, some CNT-based sensors have demonstrated a satisfactory selectivity with the help of functionalization [17,27,42,43].

#### 2.3.6. Device Structures

(i) Field effect transistor

A field-effect transistor (FET) using SWCNT(s) as the active channel is a versatile sensor platform. The simplest FET consists of two electrodes (the source and the drain), connected by a semiconductor as the channel, and a gate electrode located typically at the back of an insulating gate oxide substrate that applies a gate voltage to modulate the channel current, which provides additional means to control the current response in the channel material when it interacts with a target gas. The amplification effect of FETs makes them easy to be used as gas sensors that can detect weak signals caused by trace amounts of gases, and are expected to blaze a novel trail in the field of trace gas detection [36]. FET-structure SWCNT-based sensors commonly have a better sensitivity [30] and provide more data for sensing analysis than a resistive sensor. For example, the investigation of I–V characteristics by FET experiments is a powerful tool for probing the sensing mechanism. However, the fabrication of FETs requires advanced techniques such as lithography and controlled preparation of semiconducting SWCNTs.

(ii) Two-Electrode Sensors

The most common structure is a resistive sensor, where only two electrodes are used. In the absence of the gate electrode, this structure is simpler than transistors, leading to a low cost, making it available for widespread use. The sensors show changes in conductivity when exposed to target gases but are not suitable for further investigation of working mechanisms. Sometimes, although a sensor is fabricated with the configuration of FET, it has resistive behavior [44] due to the metal decoration on SWCNTs.

### 2.4. The Origin of the Sensing Response

**Figure 4:**
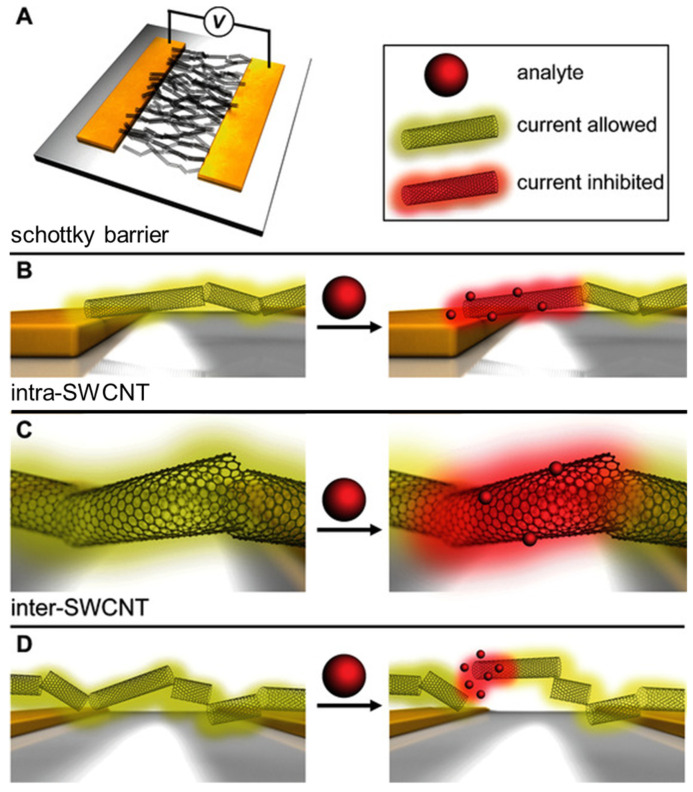
(**A**) Schematic of sensing mechanisms in SWCNT network-based resistive sensors; (**B**) at the interface between the metallic electrode and the SWCNT (Schottky barrier); (**C**) at the sidewall or along the length of the SWCNT (intra-SWCNT); (**D**) at the SWCNT−SWCNT interface (inter-SWCNT). Reproduced with permission from Ref. [37]. Copyright 2016 John Wiley and Sons.

The frontier orbitals of SWCNTs described using the band structure are better at predicting or describing the sensing mechanisms [32], according to solid-state physics. The responses of the SWCNT-based sensors (shown in Figure 4) are attributed to effects resulting from (a) contact between the tubes and the electrodes (Figure 4B, Schottky barrier modulations); (b) the sidewall or the length of the tubes (Figure 4C, intra-SWCNT); or (c) contact points between nanotubes (Figure 4D, inter-SWCNT). To some extent, all these sites can be regarded as effective sites. For sensor devices consisting of a network of SWCNTs, responses at the interfaces between nanotubes may be significant to the electronic properties of the overall network, because the distance between tubes might be changed by the gas absorption [45]. The active sites that dominate the response may differ with the analyte, the type of SWCNT, and the device structure [46].

**Figure 5:**
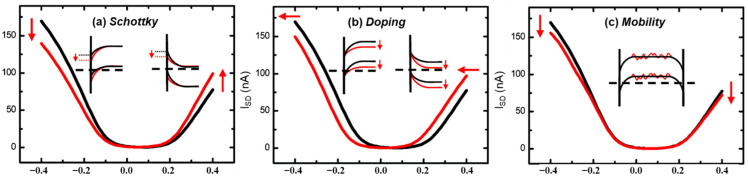
Hypothetical transfer (I_SD_−V_G_) curves before (black) and after (red) gas adsorption for three different sensing mechanisms. Insets illustrate the corresponding changes in the band diagrams: (**a**) Schottky barrier modulation corresponds to a change of barrier height, the work function difference between metal and SWCNT; (**b**) N-doping of the CNT induces a shift of the I−V curve to more negative voltages; (**c**) Change in Mobility induced by factors that reduce the conductivity, such as the addition of resistive elements or carrier scattering. Adapted with permission from Ref. [47]. Copyright 2008, American Chemical Society.

Electrical measurements on resistive sensors cannot provide sufficient information to elucidate their gas sensing behaviors, but I–V testing of SWCNT FETs can distinguish the different sensing mechanisms. When a metal contacts SWCNTs, a potential barrier arises due to the difference in their work functions, as a result of which the junction may exhibit rectifying characteristics, which is called a Schottky barrier. Under a constant bias voltage, the conductance of semiconducting SWCNTs can be changed by changing the gate voltage (V_G_), which modifies the Schottky barrier and therefore the probability of a hole (h+) traveling from the metal contact into the CNT valence band [10]. For gas sensing, the adsorption of gases may cause a change in the doping level of the material, altering the Fermi level and work function, which changes the height of the barrier [30], as shown in Figure 5a, which may be used to increase the sensitivity.

If a metal and SWCNT are in Ohmic contact, the change in the Schottky barrier is typically negligible. Intra-SWCNT sensing mechanisms are the main modes of interaction between gas molecules and individual nanotubes or nanotube bundles. Charge transfer induced directly or indirectly by interactions between gases and SWCNTs will change the conductance of the SWCNT by decreasing or increasing the concentration of the majority charge carriers. Based on the relative energy levels of the CNT and analyte, the gas molecules can act as an electron donor (n-type dopant) or acceptor (p-type dopant), respectively, shifting the threshold voltage to a smaller or larger value [32] (Figure 5b).

If the transfer curve shows a lower conductance in both the p- and n-branches (Figure 5c), it means a reduction in the charge carrier mobility by charge carrier trapping or scattering. Any disturbance of the ideal SWCNT structure introduces charge scattering sites.

In fact, the measured curves from FET sensors are rather complicated because of the coexistence of these three factors. However, it is possible to distinguish the dominant sensing behavior by passivating different areas of the constructed FET sensors. For example, Bradley et al. [48] showed that the dominant NH_3_ sensing area is the CNT channel and not the CNT/electrode interface by comparing the response of sensors with and without passivated metal-nanotube contacts. Liu et al. [49] reported that both the center and contact regions function in gas sensing by exposing the center, or contact region, of CNT devices to oxidizing or reducing gases. Zhang et al. [50] covered CNT/electrode contacts with a thick and long passivation layer that prevented their direct exposure to the gas and found a considerably delayed response that was consistent with the diffusion of the gas through the passivation layer, showing that CNT/electrode interfaces dominate the response. Peng et al. [51] suggested that changing the Schottky barrier at the contacts is the dominant mechanism from room temperature to 150 °C by comparing three CNT-based FET structures changed by without and with passivation of the CNT/electrode contacts or the CNT channel with a Si_3_N_4_ layer. For practical sensors, all three sensing methods should be taken into consideration to achieve the desired performance.

## 3. Approaches to and Progress in Improving the Sensing Performance

Although pure SWCNT-based gas sensors showed excellent ability in detecting electron acceptor or donor molecules, their poor selectivity and low affinity to some target gases such as CH_4_ and H_2_ have prevented their widespread use. As a consequence, many approaches, which can be classified into sensor device design and active material optimization, have been developed to improve gas sensing performance. In this section, we will focus more on the materials aspect. For details about the design of sensor configurations, please refer to some previous review articles [52,53,54,55].

### 3.1. Pure SWCNT-Based Sensors Produced by Tuning the Structure

A pure SWCNT-based sensor is able to detect target gases at trace concentrations, and the sensing performance varies with the source of the SWCNTs [56] even with same the pre-treatment. The quantity and morphology of SWCNTs play an important role in determining the gas sensing performance [32,57], with parameters such as tube density (from individual to networks), an isolated or bundled state, type and concentration of defects, and amounts of metallic or semiconducting tubes need to be taken into consideration.

#### 3.1.1. Individual Tubes or a Network Sensor

Individual SWCNTs are an ideal material for investigating sensing mechanisms because they eliminate the need to separate intra-tube and inter-tube interactions [32]. Devices using single SWCNTs have a higher sensitivity and lower LOD than those using bunches or networks, as the former can sense the change produced by a single chemical event or the presence of a single molecule [39,40,58,59,60]. However, the fabrication and characterization of individual SWCNT sensors is very time-consuming and needs expensive equipment to detect the small signals with high precision. Furthermore, the device-to-device reproducibility remains a challenge due to the SWCNT-to-SWCNT variations including an uncontrollable chirality [61,62,63].

An SWCNT network is an alternative to a sensor using individual SWCNTs and is much simpler, faster and more economical. For example, the continuous fabrication of meter-scale SWCNT films [64,65] using a floating catalyst chemical vapor deposition (FCCVD) method has been reported, showing the potential for the mass production of flexible, high-quality and conductive SWCNT networks at a low cost. An SWCNT network gives a higher device-to-device reproducibility compared to devices using individual SWCNTs. However, new parameters of a network including junctions between tubes, and the density of the tubes must be taken into consideration, because they are reported to have an important effect on the sensing performance. Barbara et al. [46] found that junctions in a network dominate the response when there is a high density of them. However, when the number of junctions decreases, as in the case of low-density networks, the electrodes start playing a substantial role in the response and eventually become the main response mechanism for single-CNT devices, where no CNT junctions exist. The density of SWCNTs also has an effect on the sensing performance. Ishikawa et al. [66] constructed a biosensor using films with a low density of SWCNTs with a high surface-to-volume ratio, which demonstrated good sensitivity and low LOD. Our group found that sensors constructed using networks with different densities of SWCNTs showed different H_2_ responsivities, verifying the importance of the number of SWCNTs per network area for gas sensing [20].

**Figure 6:**
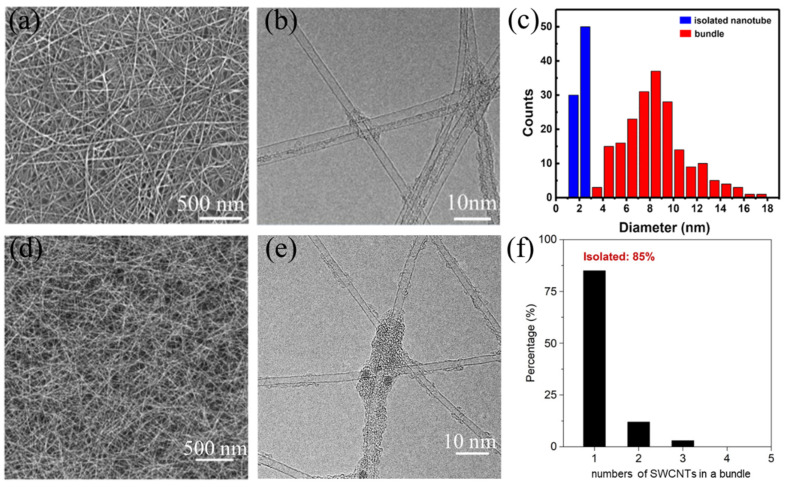
Microstructures of small-bundle [74] SWCNTs (**a**–**c**) and isolated [75] SWCNTs (**d**–**f**) with carbon-welded joints. (**a**,**d**) Typical SEM images. (**b**,**e**) Typical TEM images. (**c**,**f**) Statistical data of the numbers of isolated and bundled SWCNTs in the network. Reproduced with permission from Ref. [74]. Copyright 2018 Elsevier, and Reproduced with permission from Ref. [75]. Copyright 2018, American Association for the Advancement of Sciences.

SWCNTs usually aggregate into large bundles due to the strong van der Waals force between adjacent tubes. Investigations on the gas sensing mechanism of SWCNT bundles [67,68] have been performed, and the origin was attributed to gas adsorption on interstitial channels [69,70] or confinement effects [71]. To achieve good intra-CNT sensing, bundled SWCNTs should be avoided, because de-bundling produces higher responses and sensitivities [72,73]. Recently, high-quality small-bundle [74] and isolated [75] SWCNT films (Figure 6) were directly synthesized by an FCCVD method. Compared to the common SWNCTs in large bundles, a much larger number of charge carriers remain accessible [76] to gas interactions and/or to be coupled to receptor groups [77] due to the large exposed surface area of isolated/small-bundle SWCNTs [72]. In addition, carbon welding in the junctions of isolated SWCNTs forms Ohmic contact, making it an ideal material for the investigation of intra-tube sensing mechanisms.

#### 3.1.2. Quality and Defects

It is well known that defects including pentagons, heptagons, vacancies, or dopants inevitably exist in SWCNTs, and these can drastically modify the electronic properties [78]. Those defect sites serve as both low-energy adsorption sites and nucleation sites for additional condensation of the gas molecules on the surface of SWCNTs and the charge transfer occurs primarily when an adsorbate binds to a defect site, causing a change in resistance [29,79]. Because the resistance of a nanotube changes by three orders of magnitudes when defects form on its surface [80], Salehi-Khojin et al. [81] found that the sensing behavior largely depends on bottlenecks in the conduction paths. For highly conductive SWCNTs, the sensing was dominated by the junctions between the nanotubes as well as the electrode-nanotube junctions. For less conductive SWCNTs, the sensing was dominated by the tubes (intra-tube interactions), due to a large resistance caused by the presence of defects.

Perkins et al. [79] found that adsorption at defect sites produces a larger response due to there being an increased adsorbate binding energy and more charge transfer at defect sites by a combination of electronic calculations and experimental data, as well as oxidation of ~2% of the carbon atoms leading to a significant decrease in electrical conductivity. They emphasized that the controlled introduction of defects could increase the sensitivity and chemical selectivity of the conductance response [79]. Byun et al. [82] intentionally induced defects on the surface of s-SWCNTs by rapid thermal annealing in an Ar atmosphere. They found that sensors fabricated from these CNTs had a much higher sensitivity than those formed from the original s-SWCNTs. They also [83] fabricated a sensor using these defective SWCNTs decorated with N-[3-(trimethoxysilyl) propyl]ethylene diamine (en-APTAS) molecules, which showed high sensitivity and selectivity toward NO. Yu et al. [84] used first-principles density functional calculations to simulate the structural and electronic properties of SWCNTs after the physical and chemical adsorption of molecular and atomic hydrogen, oxygen and nitrogen on vacancy defects. They found an interesting phenomenon in which defective SWCNTs showed half-metal properties and molecular chemisorption converts them back to a semiconductor.

At present, there is no research that clearly shows which type of defect is best for improving sensing performance. However, it is clear that there is an optimum density of defects to give the best sensing performance. Too many defects greatly decrease the electron transport ability of SWCNTs, increase the work function, and change those that are semiconducting to half-metallic ones [84], thus decreasing the sensor performance. As a result, a balance must be made between defect concentration and the intrinsic electronic properties of SWCNTs to give optimal performance [32].

#### 3.1.3. Electrical Conductivity type of SWCNTs

Currently, the performance of SWCNT thin film transistors is limited by the coexistence of both metallic and semiconducting nanotubes, resulting in a high off-state leakage current and low on/off ratios [85,86]. TFT sensors fabricated using a film of unsorted SWCNTs are less sensitive to analytes than one using an s-SWCNT-enriched film. The sensing response is lower because of a reduced density of states near the Fermi level in m-SWCNTs compared with the valence band edge of s-SWCNTs [76].

**Figure 7:**
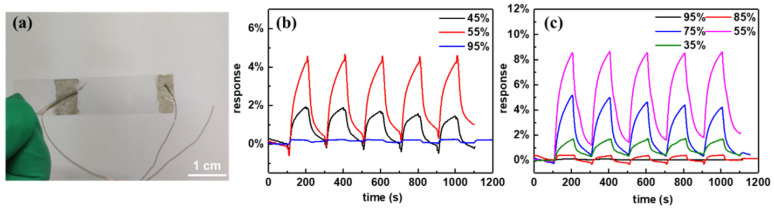
(**a**) Optical images of a hydrogen gas sensor constructed using SWCNT films with a transparency of 85%. Responses of (**b**) m-SWCNT and (**c**) s-SWCNT films with different transparencies upon exposure to H_2_ with a concentration of 5% (vol.). Reproduced with permission from Ref. [20]. Copyright 2019 Elsevier.

Our group [20] fabricated a flexible and transparent hydrogen sensor using high-quality semiconducting or metallic-enriched SWCNT films as sensing materials [Figure 7a]. It was found that all the s-SWCNT-based resistive sensors constructed with different film thicknesses exhibited a much better sensing performance than the metallic counterpart (Figure 7b,c). Agarwal et al. [87] constructed aligned s-SWCNT-based resistive devices by shadow mask in situ sorting with 4 wt% sodium dodecyl sulfate (SDS). A sensitivity improvement of ~21 to 76% in the 20–80 ppm NO_2_ concentration range was observed in the case of aligned s-SWCNT devices compared to the random network-based sensors. Thereafter, a sorted high concentration of s-SWCNTs has been used extensively as a sensing material [88,89,90,91,92]. Specifically, a floating gate FET-structured sensor constructed using a high-purity network film of s-SWCNTs decorated with Pd nanoparticles as the channel [88], showed a record LOD of 90 ppb at room temperature and even reached the sub ppb level at higher temperatures.

### 3.2. Functionalization of SWCNTs

As described above, much progress has been made in SWCNT-based gas sensors by controlling the network structure, defects, and conductivity type of SWCNTs. However, the inertness of sp^2^ carbon makes a pure SWCNT-based sensor have a low sensitivity for analytes such as H_2_, CH_4_, and CO_2_ [6,93]. Furthermore, it is difficult to selectively detect a target gas in a gas mixture using pure SWCNT sensors. In order to improve the sensing performance, receptors that selectively recognize, interact or react with the target gas are commonly anchored on the surface or ends of the SWCNTs. Various methods have been proposed to modify SWCNTs, which can be classified as covalent and noncovalent functionalization [94]. If the receptor reacts to form a covalent bond with the SWCNTs it’s called covalent functionalization. Covalent functionalization is strong and stable but lowers the intrinsic electronic properties of SWCNTs [32]. Therefore, the degree of functionalization must be carefully controlled to achieve an optimum result. Noncovalent functionalization mainly involves the absorption of molecules containing receptors which are attractive because they produce less perturbation to the intrinsic properties of SWCNTs. The drawback of noncovalent functionalization is that it is not stable, which limits the working conditions of the sensor.

#### 3.2.1. Covalent Functionalization

**Figure 8:**
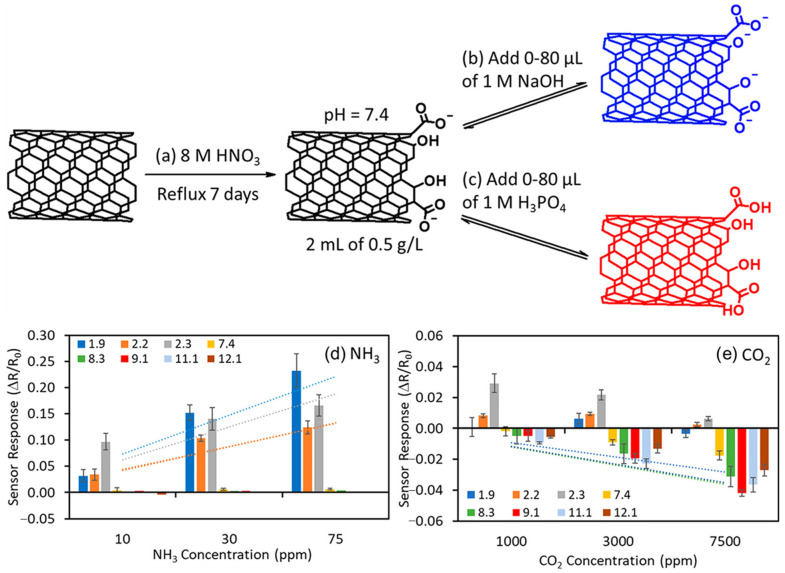
(**a**) Oxidation of SWCNTs. (**b**) Addition of base to SWCNT−COOH solution. (**c**) Addition of acid to SWCNT−COOH solution. (**d**,**e**) Sensor responses (ΔR/R_0_) of acid- or base-pretreated SWCNT−COOH samples to (**d**) NH_3_ and (**e**) CO_2_. Data for each bar is averaged from three different gas exposures. Reprinted with permission from Ref. [95]. Copyright 2019, American Chemical Society.

Covalent modification generally disturbs the π-electron system and adds defects, while increasing the stability of an SWCNT dispersion [37]. Common covalent functionalization uses strong oxidation to introduce carboxylic acid (−COOH) and hydroxy (−OH) moieties at defect sites. As shown in Figure 8a, SWCNTs might be cut short by heavy oxidation, and −COOH or −OH functional groups are covalently bonded at the ends of the CNTs. Adding an acid or base to the carboxylation process change the pH of the resulting material and yield inks with varying pH values (Figure 8b,c). Kim et al. fabricated 8 carboxylated (−COOH) SWCNT sensors [95] with controlled pH values in the range of 1.9–12.1 for NH_3_ and CO_2_ detection. The sensors responded to various levels of NH_3_ and CO_2_ at ambient temperature (Figure 8d,e). At pH 1.9, the sensor was 40 times more sensitive to NH_3_ than one using a nonconditioned SWCNT−COOH (pH 7.4) sample. At pH 9.1, the sensor achieved 2 times more sensitivity to CO_2_ compared to the nonconditioned case. They [96] also demonstrated an array of sensors that gives orthogonal responses to target gases and vapors from acidic (CO_2_, H_2_S, HCl, and HF) to basic [NH_3_,CH_3_NH_2_, (CH_3_)_2_NH, and (CH_3_)_3_N] gases, by controlling the pH during carboxylation. These functional groups have the ability to covalently link to other molecules containing receptors by the formation of amide or ester bonds [94]. Haddon et al. [97] covalently bonded poly(m-aminobenzene sulfonic acid) (PABS) to carboxylated SWCNTs with the help of oxalyl chloride, and fabricated SWCNT−PABS based sensors [98]. The LODs of the sensor for NH_3_ and NO_2_ were 100 and 20 ppb, respectively, at room temperature with a short response time.

Apart from at the ends of SWCNTs, covalent functionalization can happen in the side wall [99]. Side-wall reactions include dipolar cycloaddition [100], reductive reactions with diazonium ions [101,102,103], and other complicated methods [104,105]. For example, Hassan et al. [100] covalently functionalized SWCNTs for ammonia gas sensing with 1,6-diethynylpyrene by an azide-alkyne Huisgen cycloaddition reaction. The sensor showed a LOD of 0.5 ppm.

**Figure 9:**
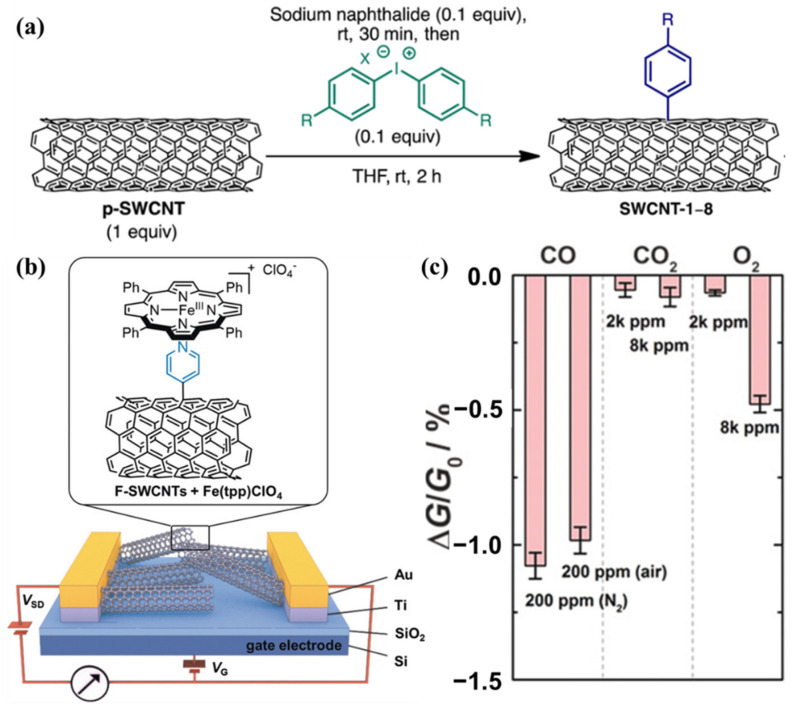
(**a**) Schematic of the covalent functionalization of CNTs by iodonium salts. Reprinted with permission from [106], Copyright 2016, American Chemical Society. (**b**) Bio-inspired carbon monoxide sensor. Schematic of a FET substrate with source-drain electrodes, a SiO_2_ dielectric layer and a Si gate electrode. Chemical structures of a pyridyl-functionalized SWCNT and iron porphyrin (Fe−(tpp) ClO_4_), describing the coordination of the pyridyl group to the iron center of the porphyrin. (**c**) Comparison of the responses to CO in both N_2_ and air to the responses to CO_2_ and O_2_. Reproduced with permission from Ref. [42]. Copyright 2017 John Wiley and Sons.

Timothy and coworkers [106] reported an efficient method for the covalent functionalization of CNTs by iodonium salts, by precisely attaching single aromatic rings to the sidewalls of SWCNTs (Figure 9a). They then fabricated a hemeprotein-inspired sensor [42] for carbon monoxide using pyridyl-functionalized SWCNTs and iron porphyrin (Fe−(tpp) ClO_4_) with the iodonium ions as shown in Figure 9b, which exhibited a detection ability toward ppm levels of CO in air with highly specific responses (Figure 9c). However, this type of method requires complex organic chemistry to design the reaction to obtain the desired functionalization.

#### 3.2.2. Noncovalent Functionalization

Noncovalent modification is less invasive than covalent functionalization as it relies on π interactions and van der Waals interactions between SWCNTs and molecules [37] which could be directly used to sense molecules at trace concentrations [59,107]. SWCNTs can also be noncovalently functionalized by the physisorption of aromatic molecules and surfactants [99] by π−π stacking. Hydrophobic interactions between SWCNTs and polymers with surfactant characteristics can drive their solubility in water, and saccharides and polysaccharides are capable of solubilizing and functionalizing SWCNTs. [32] This method is commonly used [108,109,110] to adsorb proteins for biosensing. Novel 1D van der Waals heterostructures, which could be considered a new type of noncovalently functionalized SWCNTs were recently reported by Xiang et al. [111,112,113]. The electronic and optoelectronic devices constructed using these novel van der Waals heterostructures showed excellent performance. Such functionalized SWCNTs may also find application in gas sensing due to their designable and tunable noncovalent bonding and electronic structures.

**Figure 10:**
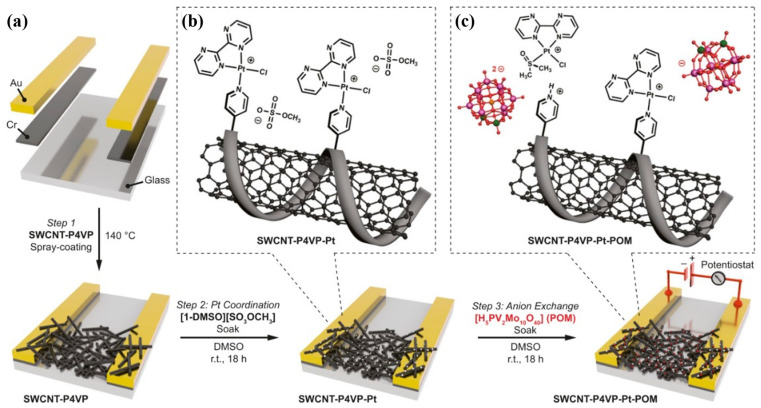
Diagram of device fabrication and sensing element composition. (**a**) Sensor fabrication including spray coating of an SWCNT−P4VP network, Pt coordination and anion exchange (**b**,**c**). Proposed surface speciation of (**b**) SWCNT−P4VP−Pt and (**c**) SWCNT−P4VP−Pt−POM. Reproduced with permission from Ref. [43]. Copyright 2021 National Academy of Sciences.

Datta et al. [114] noncovalently functionalized SWCNTs with poly(N-methyl pyrrole) (P[NMP]) by π−π interaction, with the product having an excellent linear response from 10 ppb to 1 ppm for ammonia sensing. Pankaj et al. [17] reported a flexible NO_2_ sensor based on polyethyleneimine-coated SWCNTs, which showed a room temperature high sensitivity to NO_2_ gas in dry air in the range from 0.75 ppm to 5 ppm. The sensor was almost insensitive to ammonia, demonstrating high selectivity. Recently, a room temperature methane sensor was reported by Swager’s group [43]. The chemiresistor was based on SWCNTs noncovalently functionalized with poly(4-vinylpyridine) (P4VP) that enabled the incorporation of a platinum-polyoxometalate (Pt−POM) CH_4_ oxidation precatalyst into the sensor by P4VP coordination. The first step of the functionalization is the pyridyl lone pair−π and π−π interactions between the SWCNT and P4VP, as illustrated in Figure 10a. In addition, free pyridyl groups in P4VP can be used to coordinating the Pt, as shown in Figure 10b. Finally, a Pt−POM CH_4_ oxidation precatalyst was achieved by anion exchange (Figure 10c). The chemiresistors showed a ppm level sensitivity to CH_4_ and good air and moisture stability, as well as selectivity for methane over heavier hydrocarbons and carbon dioxide. Bezdek et al. [19] fabricated flexible SWCNT sensors using a similar method, which could operate at room temperature with a low power requirement, potentially suitable for wearable sensors or for the rapid in-field detection of trace H_2_S. Liu et al. [115] constructed a chemiresistive CO sensor based on SWCNTs noncovalently functionalized with Cp^˄^CoI_2_, an organocobalt complex with an intramolecular amino ligand coordinated to a metal center that is displaced upon CO binding. The resulting device showed ppm-level LOD and unprecedented selectivity for CO gas among CNT-based chemiresistors.

Due to the weak binding of noncovalent functionalization, the operating conditions of the sensors must be mild. However, a common challenge for both the covalent and noncovalent functionalization is to know which effective receptors to use to obtain the best response with specific gases.

#### 3.2.3. Decoration with Nanoparticles

**Figure 11:**
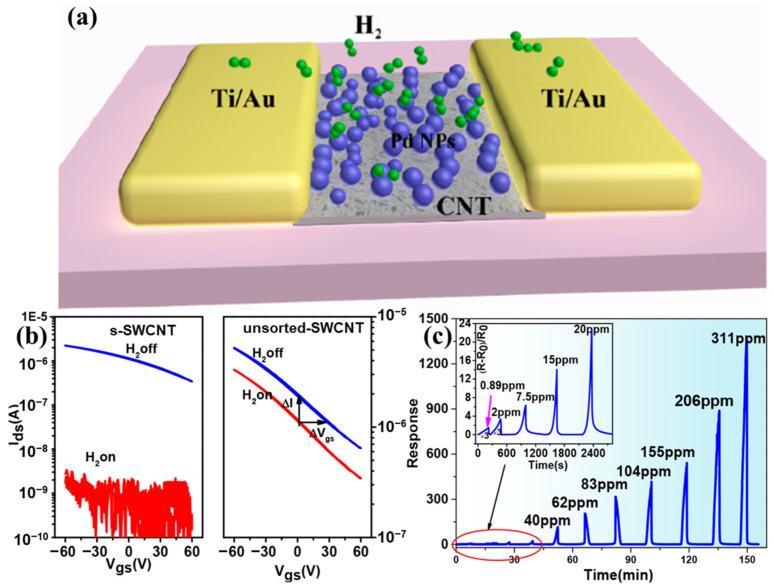
(**a**) Schematic of a H_2_ sensor based on a SWCNT film decorated with Pd. (**b**) Transfer curves of the sensors based on s-SWCNTs (left) and unsorted-SWCNTs (right) before (blue) and after (red) 311 ppm of H_2_ exposure for 200 s. (**c**) Real-time response to different H_2_ concentrations at room temperature. Inset: magnified plot at low concentrations. Reprinted with permission from Ref. [44]. Copyright 2018, American Chemical Society.

Although the decoration of SWCNTs with nanoparticles has been classed as noncovalent functionalization by some researchers [94], we prefer to list it separately. Nanoparticles with a high affinity for the target gas molecules have been used to improve the sensitivity, selectivity, LOD, as well as response times of SWCNT detectors. Physical depositions such as electron-beam evaporation [44] and sputtering [116] of the nanoparticles are the most popular methods, but chemical functionalization with nanoparticles can be achieved by hydrothermal and sol-gel methods [117]. The reported nanoparticles include metals such as Pt [118,119], Pd [120,121,122,123],Au [124,125,126,127], Ag [128,129,130], Cu [131,132], Ru [133], and metal oxides [56,134,135,136,137,138]. Taking Pd as an example, when H_2_ gas molecules contact Pd, H_2_ dissociates on the surface and forms PdH_x_ [139,140], which promotes electron donation to compensate for hole carriers in the SWCNTs and leads to higher resistance. As a result, Pd nanoparticles have served as great receptors for CNT-based sensors to selectively sense hydrogen. Reginald [141] et al. fabricated H_2_ resistive sensors using Pd nanoparticle-decorated CNT ropes as the sensing element, which showed a high response and a LOD down to 10 ppm. More recently, Peng et al. [44] developed a large-scale method to fabricate ultrasensitive FET type H_2_ sensors based on solution-sorted s-SWCNTs decorated with Pd nanoparticles (Figure 11a). Ti contacts were used to form a Schottky barrier (Figure 11b) to improve charge transfer giving rise to the resistance change of the sensor. The fabricated sensors had a very fast response time of 7 s at 311 ppm and a detection limit of 890 ppb (Figure 11c), which is the highest response to date for resistor-based sensors and were the first to have sub-ppm detection for H_2_ at room temperature.

**Figure 12:**
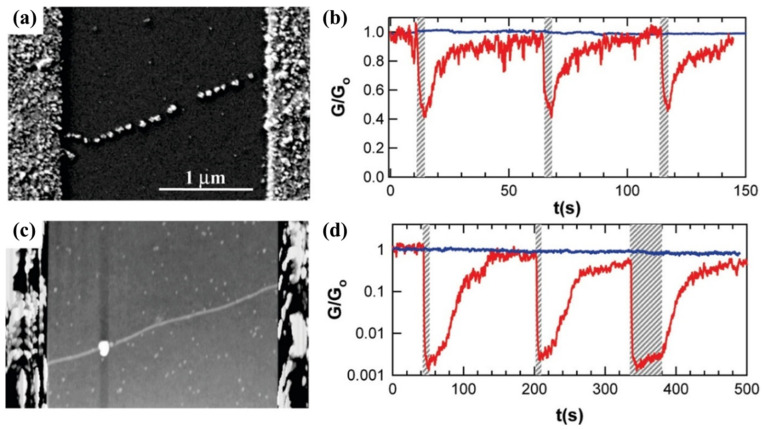
(**a**) SEM image of an s-SWCNT device with random Pd decorations all along its length. (**b**) Response of the device to pulses of H_2_ in air, before (blue) and after (red) the Pd deposition. (**c**) Atomic force topography image of a second SWCNT with a production of a point defect and the selective decoration of these sites with Pd. (**d**) Response of the device with a point defect, before (blue) and after (red) Pd deposition, showing the nearly thousand-fold better response. Reprinted with permission from Ref. [142]. Copyright 2010, American Chemical Society.

Collins et al. [142] emphasized the importance of the particle location in decorated SWCNTs. They made two sensor devices using individual SWCNTs. One was constructed using a relatively perfect SWCNT randomly decorated with Pd, as illustrated in Figure 12a. The other was fabricated using an SWCNT containing a point defect and selectively decorated with Pd at this defective site (Figure 12c). The latter showed an almost thousand-fold increase in resistance, demonstrating complex interdependence between a defect site’s electronic transmission and the chemistry of the defect-Pd-H_2_ system [142].

As mentioned in the section on quality and defects, the number of defects must be controlled. The functionalization of CNTs can also be regarded as a useful or effective “defect”. Thus, to achieve the optimum sensing performance, the ability to control the concentration and position of any functionalization is of great importance.

## 4. Challenges and Outlook

Notable progress has been made on SWCNT-based gas sensors in recent years, and their performance including sensitivity, response time, LOD, and selectivity show great promise for future commercial application. However, there are still many challenges to overcome when cost, reproducibility, environment compatibility, and scaling-up are taken into consideration. Since the performance of a sensor is mainly determined by both the active sensing material and device structure, we point out the following two major challenges.

From the aspect of the sensing material, individual SWCNTs have shown the ability to detect extremely low concentrations of analytes, even a single molecule, and are also ideal for investigating the sensing mechanism of SWCNTs. However, expensive equipment and complex fabricating procedures for the devices hinder their large-scale commercial use. SWCNT networks seem to be a good choice for constructing simple and low-cost sensors. The SWCNT network should be comprised of isolated nanotubes to fully expose the C atoms, and high-purity semiconducting SWCNTs are needed for their sensitive resistance changes when target molecules are adsorbed. Improving the selectivity of SWCNT-based sensors is a big challenge, which it seems could be solved by functionalization with suitable receptors. This depends not only on the design of efficient receptors for the target gas, but also on establishing suitable functionalizing methods together with a well-controlled degree of functionalization to obtain better sensing performance. The most important challenge is to work out the homogeneity and reproducibility of SWCNT-based gas sensors, which is the requirement for industrialization.

From the device point of view, the selectivity issue can be addressed by the structural design of the device. For example, we can design sensor arrays or multi-channel detectors to recognize the fingerprints of different gases. The basic principle is that in response to a target gas, a sensor array puts out signals that form a unique fingerprint for that specific gas [27]. Every sensor unit with different SWCNT functionalizations would show a different response to an analyte gas, and thus gas libraries can be established to distinguish them. Artificial intelligence and machine learning [94,95,140] can be used to improve the accuracy of gas detection. Last but not least, the booming IoT sets higher requirements for next-generation sensors. Flexible or wearable sensors [17,141,142], and those with ultralow power consumption or even powerless (self-powered), and the ability to wirelessly communicate [139,143,144,145,146], and combinations of these [147,148,149] need to be considered in the future device fabrication.

## 5. Conclusions

SWCNTs are one of the most promising materials for fabricating flexible gas sensors due to their unique geometries and extraordinary intrinsic properties, as well as their ability to be tailored to detect target gas molecules. SWCNT-based gas sensors have shown an excellent sensing performance including a fast response time and an extremely low LOD at room temperature. In this article, we have summarized the sensing principles, important parameters, and state-of-the-art research progress of SWCNT-based gas sensors. Possible approaches for improving the sensing performance from the materials aspects have been discussed. Considering the cost and efficiency, SWCNT networks comprised of isolated s-SWCNTs with an appropriate degree of functionalization could be a promising candidate sensing material. Combining the design of sensor structure and configuration with SWCNT flexibility and transparency, SWCNT-based gas sensors may have great promise for use in the IoT, wearable devices and aerospace applications.

## Data Availability

Not applicable.
